# Complicated Rupture of Urethra

**Published:** 1887-02

**Authors:** Rudolph Menger

**Affiliations:** San Antonio, Texas


### COMPLICATED RUPTURE OF URETHRA.

*By Dr. Rudolph Menger, of San Antonio, Texas.*

Reported for Daniel's Texas Medical Journal.

BELIEVING that the following case is a rare one, and of great interest in some respects, I report the particulars:

J. B., aged about 45, of robust and healthy appearance, weighing nearly 200 pounds, undertook early one morning of last month to catch a turkey on the roof of a house. Unluckily the man slipped and fell, with legs stretched apart, on the edge of the upper board of a fence and directly on his perineum and scrotum. He had taken a drink of whisky just before the accident happened, and had emptied his bladder before he ascended the fence to get on the house. When first seen, in company with Dr. Hertzberg, we found the pars bulbosa swollen and slight discoloration of the perineum and scrotum. He did not complain much, except on pressure on the perineum. The bladder was empty, and we delayed catheterism until about three hours afterwards, when I saw him again and found the bladder filling and blood oozing from the meatus urethræ. I tried to introduce a soft catheter, of course as cautiously as possible, but failed. After consulting with Dr. D. Berry, we again tried to introduce a soft catheter, bougie or metallic catheter, but in vain. After each of these trials, a large quantity of blood was discharged by the catheter, and it was evident that the urethra was ruptured at the pars membrana, and also that either old strictures or false passages, caused by the traumatism, existed, as the catheter would pass one large and a smaller obstruction, but would not enter the bladder.

As the man was in a very small room of a boarding-house with but little light and devoid of the least necessary attention, and where it would have been nearly impossible to make any sort of operation, we had him removed to the Hospital. At this time his bladder was filled to the utmost. After bringing him fully under chloroform, I again tried catheterism; first with soft catheter, then with a stricture sound, and requested Dr. A. Herff and others to see if they could be more successful—but without success. As I had no large curved trocar at hand, I aspirated the bladder through the rectum, which of course, was only a temporary relief; and, as the bladder filled up again during the night of the following day, I passed a curved trocar through the rectum in the bladder, with the intention of retaining it, until, perhaps there would be a chance to introduce a catheter *per vias naturalis* after six or eight days; but, unfortunately, after the bladder was emptied, the canula slipped the hands of an assistant, caused by the restlessness of the patient, after the narcosis, and we made at once preparations for the perineal section, Drs. Braunagel, Berry and Hertzberg, kindly assisting. Being aware that much traumatic hemorrhage, and perhaps urinary infiltration had occurred, I carefully removed clot after clot in the cut wound, which I thought, perhaps pressed on, or occluded the ruptured part of urethra. The perineum was quite broad and very deep, but the end of the staff, which lodged near the bulbus, could be reached easily after removal of the clots.

The point of interest in this case is, that, even now it was utterly impossible to enter the bladder, either from the perineal opening, or through the entire urethra or both plans combined. A part of the upper ruptured membranes could be seen, but there was such a confusion of ruptured tissues, clots and bleeding points that it was a matter of impossibility to come into the neck of the bladder; indeed, it seemed as if false passages existed leading in different directions. I dilated the wound somewhat downward into the bulbus in order to have a better view and chance to introduce an instrument into the bladder, but without success. At the same time a serious hemorrhage set in from far back, perhaps the bulbo-urethralis, and the bleeding could only be stopped with a good tampon of absorbent cotton as the tissues were torn and clotted all around the ruptured place. The scrotum, I forgot to mention, was highly infiltrated with extravasated blood, so that several large incisions had to be made. After the bleeding was controled entirely, the bladder, now filled up again to the utmost, was tapped again per rectum and the canula retained with extra appliances. The patient was doing quite well, until on the evening of the second day after the perineal section, when he complained of severe pain along the upper thigh of left leg, and I detected emphysema along all the muscles down to the knee-joint. In spite of narcotics and bandages, the pain and emphysematous swelling increased and all at once the pulse ceased to beat, his system was thrown into a state of convulsion and he died in a few minutes in a state of collapse.

There was but little rising of temperature after each operation, but he was quite nauseated, more, it seemed caused by the narcosis; no signs of peritonitis; the last attack was perhaps one of uræmia, although the patient, I must state, drank some hours before his death, contrary to all expectation and my orders, the contents of a large bottle of beer, which a friend had sent him, and a glass of which he said would generally at once stop the vomiting, as he was subject to alcoholism, and this undoubtedly brought on his restless condition and allowed access of air through some lacerated part, into the original canal and upwards into the cellular tissue along the muscles of the thigh.

I should be very glad to hear any comments, or the report of a similar case, either through the JOURNAL or by letter.

